# Mesenchymal stem cell-laden anti-inflammatory hydrogel enhances diabetic wound healing

**DOI:** 10.1038/srep18104

**Published:** 2015-12-08

**Authors:** Shixuan Chen, Junbin Shi, Min Zhang, Yinghua Chen, Xueer Wang, Lei Zhang, Zhihui Tian, Yuan Yan, Qinglin Li, Wen Zhong, Malcolm Xing, Lu Zhang, Lin Zhang

**Affiliations:** 1 Department of Histology and Embryology, School of Basic Medical Sciences, Southern Medical University, Guangzhou 510515, China; 2 Key Laboratory of Functional Proteomics of Guangdong Province, Department of Pathophysiology, Southern Medical University, Guangzhou 510515, China; 3 Departments of Mechanical Engineering, Biochemistry and Medical Genetics, University of Manitoba, and Manitoba Institute of Child Health, Winnipeg, MB R3T 2N2, Canada; 4 Department of Textile Sciences, Department of Medical Biology, University of Manitoba, Winnipeg, MB R3T 2N2, Canada

## Abstract

The purpose of this study was to permit bone marrow mesenchymal stem cells (BMSCs) to reach their full potential in the treatment of chronic wounds. A biocompatible multifunctional crosslinker based temperature sensitive hydrogel was developed to deliver BMSCs, which improve the chronic inflammation microenvironments of wounds. A detailed *in vitro* investigation found that the hydrogel is suitable for BMSC encapsulation and can promote BMSC secretion of TGF-β1 and bFGF. *In vivo*, full-thickness skin defects were made on the backs of db/db mice to mimic diabetic ulcers. It was revealed that the hydrogel can inhibit pro-inflammatory M1 macrophage expression. After hydrogel association with BMSCs treated the wound, significantly greater wound contraction was observed in the hydrogel + BMSCs group. Histology and immunohistochemistry results confirmed that this treatment contributed to the rapid healing of diabetic skin wounds by promoting granulation tissue formation, angiogenesis, extracellular matrix secretion, wound contraction, and re-epithelialization. These results show that a hydrogel laden with BMSCs may be a promising therapeutic strategy for the management of diabetic ulcers.

Diabetes has become a global public health issue and burdens our health care system^1^. Diabetic ulcers are a severe, persistent complication of diabetes and, in the most extreme cases, can lead to amputation. The complications of these poor-healing wounds include sustained chronic inflammation, decreased secretion of growth factors and disrupted vascularization^2^,^3^,^4^. Current clinical treatments such as wound dressings, hydrogels or scaffolds used individually have not achieved the desired results; therefore, more effective therapeutic approaches are urgently needed.

Bone marrow mesenchymal stem cells (BMSCs) are reported to regulate wound healing through a series of paracrine growth factors (e.g., TGF-β, FGF)^5^, and differentiate into effector cells involved in wound healing, such as keratinocytes, fibroblasts, and endothelial cells^6^, thereby accelerating wound closure^7^ and enhancing vascularization^8^, granulation tissue formation^9^ and re-epithelialization^10^. Considering the aforementioned mechanism, the active role of BMSCs in wound healing establishes the foundation for their use in treating diabetic ulcers.

The differentiation and secretion of growth factors by stem cells are regulated by a microenvironment known as the stem cell niche^11^,^12^. Hydrogels are an ideal physicochemical mimetic of natural extracellular matrix (ECM); currently, many researchers are focused on BMSC-laden hydrogels to treat skin wounds. For example, Rustad *et al*. reported that collagen-pullulan hydrogels provide a suitable microenvironment for the delivery of MSCs, which accelerates normal wound healing and promotes neovascularization^8^. Xu *et al*. reported that MSC-laden gelatin/PEG hydrogels also accelerate wound closure and re-epithelialization and enhance epidermal maturity, neovascularization, and granulation tissue formation^10^. Although these BMSC-laden hydrogels have achieved success in the wound healing of healthy skin^7^,^13^,^14^, they may not be suitable for treating diabetic ulcers. High concentrations of inflammatory cytokines are present in the inflammatory microenvironment of the ulcer, which leads to increased protease secretion, thereby resulting in the degradation or loss of growth factors secreted by the BMSCs or other effector cells^15^,^16^; moreover, this chronic inflammatory microenvironment also can impair the activity of BMSCs. We believe that if BMSC delivery strategies are to reach their full potential, the ideal hydrogel for diabetic ulcers, in addition to having good biocompatibility, should suppress the inflammatory response or inhibit protease activity.

To this end, we developed a biodegradable, multifunctional crosslinker and an n-isopropylacrylamide (NIPAM)-based, thermosensitive hydrogel to carry BMSCs to treat diabetic ulcers. The crosslinker contains an RGD-like motif that promotes cell attachment and differentiation of BMSCs^17^. We hypothesize that the hydrogel can prohibit chronic inflammation to provide a suitable environment for BMSC function and that BMSCs laden within this hydrogel can promote wound healing. In this study, we evaluated the therapeutic effects of topical administration of BMSCs laden with this thermosensitive hydrogel on chronic inflammation, wound contraction, ECM secretion, angiogenesis, re-epithelialization, hair follicle and sebaceous gland regeneration and scar formation in a diabetic ulcer model (Fig. 1).

## Results

### Synthesis and characterization of the hydrogel

**Figure 2:**
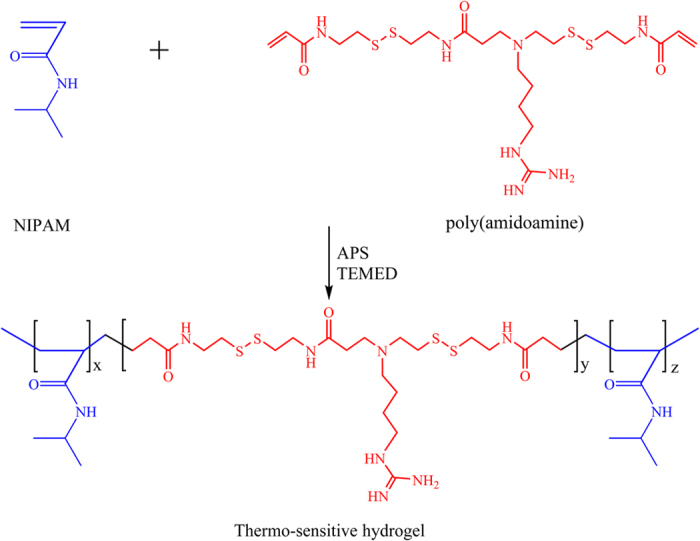
Synthesis of a PNIPAM-PAA thermosensitive hydrogel.

**Figure 3:**
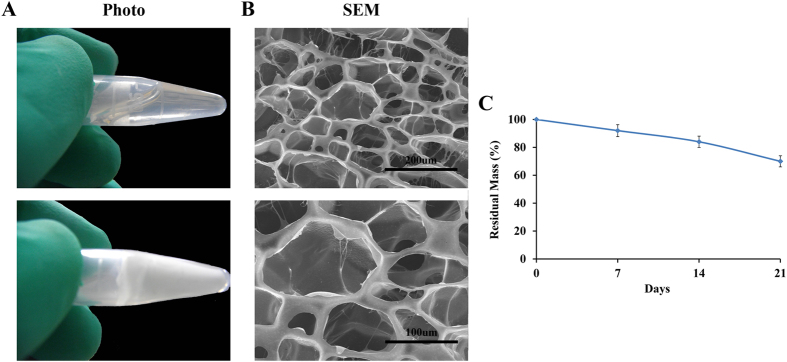
Characterization of the physical properties of the hydrogel. (**A**) The morphology and internal structure of a PNIPAM-PAA hydrogel. (**B**) The swelling behavior of the hydrogel at different hours (n = 6). (**C**) The weight loss of the hydrogel in a 10 mM DTT solution on different days (n = 6).

In this study, we employed a multi-functional crosslinker to fabricate a 3D hydrogel system by copolymerizing it with NIPAM, as shown in Fig. 2. According to Fig. 3A, the polymer presented in the sol state at room temperature; after immersion in a 33 °C water bath, it experienced sol-gel transition in minutes. Figure 3B shows the uniform porous structure of the lyophilised hydrogel. We used DTT solution to mimic a reducing environment, which plays a crucial role in disulfide bond degradation. As shown in Fig. 3C, the hydrogel was slowly degraded in DTT solution; its residual mass was (70 ± 3.99)% after 21 days (n = 6).

### Hydrogel biosecurity analysis

**Figure 4:**
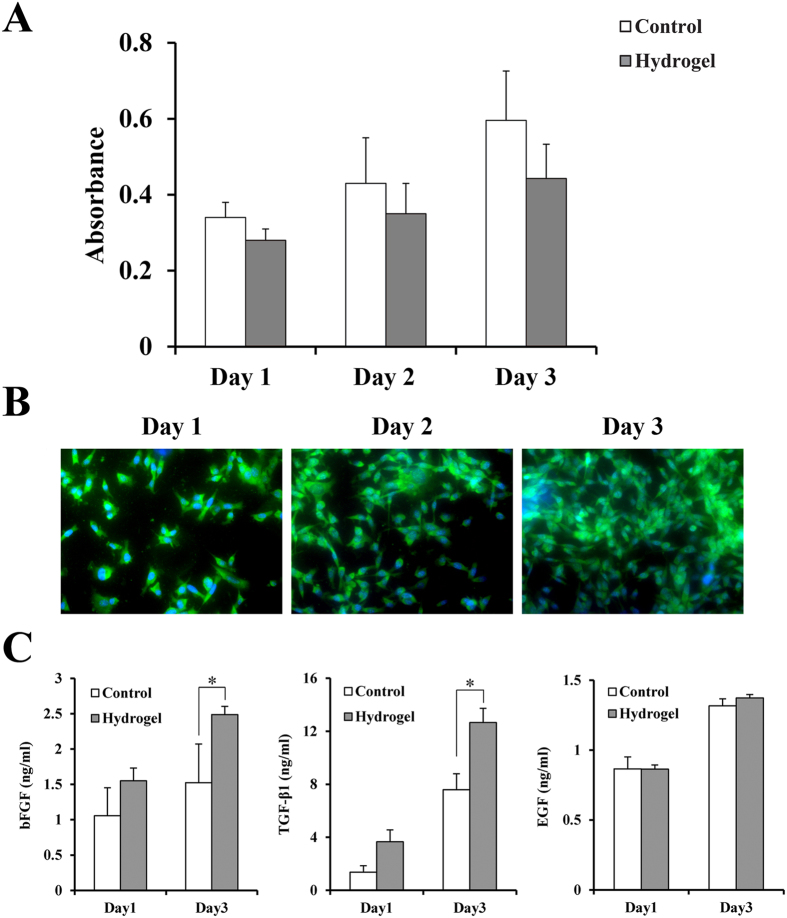
Hydrogel biocompatibility assay. (**A**) The proliferation assay of the BMSCs when cultured on the hydrogel for 1, 2, and 3 days (n = 3). (**B**) Immunofluorescence staining of BMSC proliferation on the hydrogel on days 1, 2, 3. (**C**) bFGF and TGF-β1 secretion by BMSCs when co-cultured with the hydrogel on days 1 and 3 between the control and hydrogel group (n = 6). *p < 0.05, **p < 0.01.

We seeded BMSCs on the surface of hydrogels to detect whether the hydrogel was suitable for BMSC culture. As shown in Fig. 4A,B, BMSCs can adhere to and proliferate on the hydrogel. Furthermore, the hydrogel also can promote growth factor secretion by BMSCs. As shown in Fig. 4C, on day 1, the concentrations of bFGF secreted in the hydrogel group were not higher than those of the control group on day 1; however, by day 3, there was a significant difference in these concentrations between the two groups (p < 0.05, n = 3). Similarly, the concentrations of TGF-β1 secreted by the hydrogel group were higher than those of the control group on day 3 (p < 0.05, n = 3). However, there were no differences in EGF secretion on both days 1 and 3.

### Hydrogel + BMSC combination therapy promoted wound contraction

**Figure 5:**
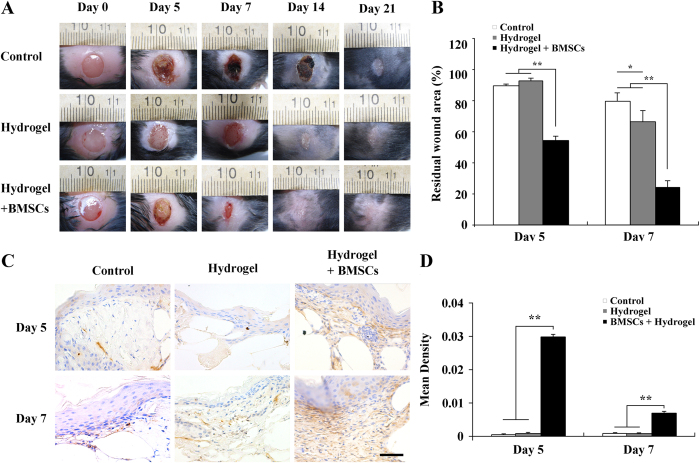
Joint hydrogel + BMSCs treatment promoted wound contraction. (**A**) General observations of the control, hydrogel, and hydrogel + BMSCs groups at different times after treatment and (**B**) comparisons of the remaining wound areas at 5 and 7 days after treatment among the control, hydrogel, and hydrogel + BMSCs groups (n = 3). (**C**) Immunohistochemical staining to detect α-SMA expression at 5 and 7 days after treatment in the control, hydrogel, and hydrogel + BMSCs groups. (**D**) α-SMA expression results at the wound site in the control, hydrogel, and hydrogel + BMSCs groups. Scale = 50 μm, *p < 0.05, **p<0.01.

We first observed the general postoperative wound healing results and calculated the residual wound area (see Fig. 5A,B). The results showed significantly greater wound contraction in the hydrogel + BMSCs group at 5 days after treatment compared with the control and hydrogel groups (p < 0.01, n = 3). Furthermore, at seven days after operation, the average unhealed area of the wound was only 24.6 ± 4.21% of the initial wound area in the hydrogel + BMSCs group, whereas the areas were 79.54 ± 5.92% and 66.5 ± 6.67% in the control and hydrogel groups, respectively.

Wound contraction resulted from the pulling effects of myofibroblasts, which express large amounts of α-SMA. Therefore, we investigated α-SMA expression at the wound sites (see Fig. 5C). Quantitative analyses of α-SMA expression for each group revealed that the hydrogel + BMSCs group exhibited significantly greater α-SMA expression than the control and hydrogel groups on both days 5 and 7 (p < 0.01, n = 3), whereas significant differences were not found with respect to α-SMA expression levels between the control and hydrogel groups (Fig. 5D).

### The hydrogel inhibited chronic inflammation

**Figure 6:**
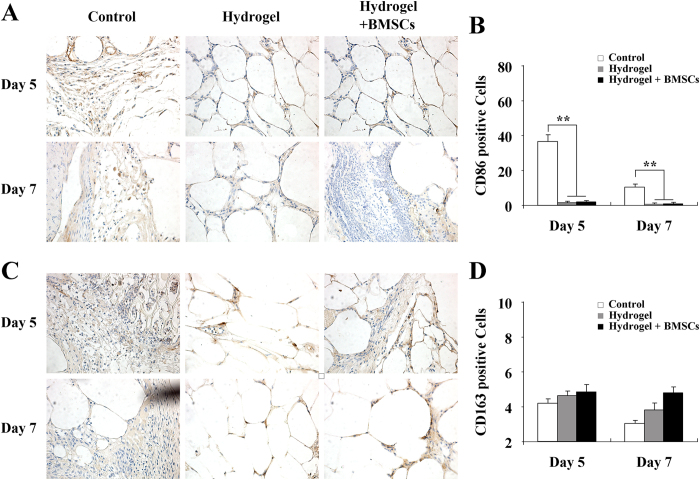
Hydrogel treatment inhibited chronic inflammatory responses at wound sites. (**A**) Immuno-histochemical detection of CD86-positive cells at wound sites on days 5 and 7 in the control, hydrogel, and hydrogel + BMSCs groups. (**B**) Statistical analysis of CD86-positive cells (n = 5). (**C**) Immunohistochemical detection of CD163-positive cells at wound sites on day 5 and 7 in the control, hydrogel, and hydrogel + BMSCs groups. (**D**) Statistical analysis of CD163-positive cells (n = 5). Scale = 50 μm. *p<0.05, **p<0.01.

Macrophages, the major inflammatory cells present in wounds, have two phenotypes: M1 and M2. M2 macrophages are necessary for angiogenesis and wound healing, in contrast to pro-inflammatory M1 macrophages; thus we detected the expression of M1 and M2 macrophages. In this study, we used CD86 to mark M1 macrophages and CD163 to mark M2 macrophages^18^,^19^. As shown in Fig. 6A,B, the level of CD86-positive cells (M1 macrophage) in the control group was markedly higher than in the hydrogel and hydrogel + BMSCs groups (p < 0.01) on both days 5 and 7, whereas there was no significant difference between the hydrogel and hydrogel + BMSCs groups. Additionally, as shown in Fig. 6C,D, there was no significant difference in CD163 (M2 macrophage) expression among the control, hydrogel, and hydrogel + BMSCs groups on both days 5 and 7.

### Hydrogel + BMSCs combination therapy promoted the formation of granulation tissue

**Figure 7:**
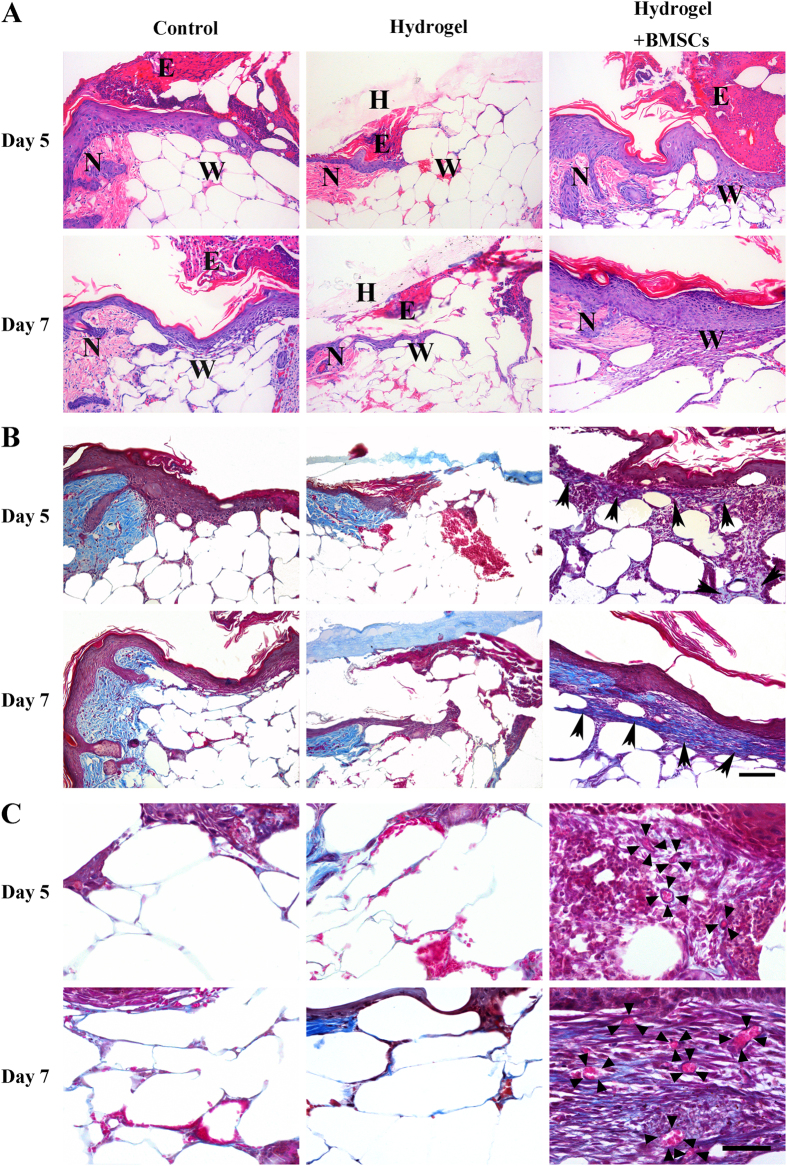
Joint hydrogel + BMSCs treatment promoted the formation of granulation tissue. (**A**) HE staining of the wound at 5 and 7 days after treatment in the control, hydrogel, and hydrogel + BMSCs groups. N: normal tissue, W: wound tissue, E: eschar, H: hydrogel. Scale = 100 μm. (**B**) Masson trichrome staining to detect the secretion of nascent collagen (black arrows) at 5 and 7 days after treatment in the control, hydrogel, and hydrogel + BMSCs groups. Scale = 100 μm. (**C**) Masson trichrome staining detection of nascent blood vessels (black triangles) at 5 and 7 days after treatment in the control, hydrogel, and hydrogel + BMSCs groups. Scale = 50 μm.

As shown in Fig. 7A, five days after treatment, in contrast to the control and hydrogel groups, histological observations revealed that the hydrogel + BMSCs group exhibited significant formation of granulation tissue at the wound site, which featured a loose structure with numerous cells found within the tissue. Seven days after treatment, more granulation tissue had formed in the hydrogel + BMSCs group, and its structure had become denser. In contrast, no significant granulation tissue had formed in the control or hydrogel groups on both days 5 and 7.

We performed Masson trichrome staining to detect the newborn collagen fibres and new blood vessels within granulation tissue. Five days after treatment, the hydrogel + BMSCs group had begun to form a small number of collagen fibres compared with the control and hydrogel groups (see arrows, Fig. 7B), and more collagen fibres had formed by day 7. In addition, new blood vessels were observed within the granulation tissue in the hydrogel + BMSCs group on day 5 (as indicated by the triangle in Fig. 7C), and blood had flowed into these cavities. Seven days after treatment, the density of new vessels had increased. However, in the control and hydrogel groups, only trace quantities of new collagen fibres were found. Moreover, the growth rate and density of the regenerated blood vessels were significantly lower in these groups compared with the hydrogel + BMSCs group.

### Hydrogel + BMSCs combination therapy promoted keratinocyte proliferation and differentiation

**Figure 8:**
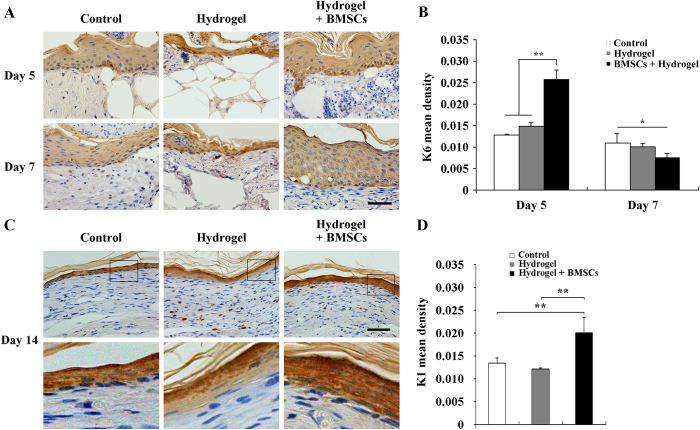
Joint hydrogel + BMSCs treatment promoted keratinocyte proliferation and differentiation. (**A**) Immunohistochemical staining of K6 expression at the wound edges 5 and 7 days after treatment in the control, hydrogel, and hydrogel + BMSCs groups. (**B**) K6 expression results at the wound edges in the control, hydrogel, and hydrogel + BMSCs groups. (**C**) Immunohistochemical staining of K1 expression at the wound site 14 days after treatment in the control, hydrogel, and hydrogel + BMSCs groups. (**D**) K1 expression results at the wound site in the control, hydrogel, and hydrogel + BMSCs groups. Scale = 50 μm, *p<0.05, **p<0.01.

The proliferation and differentiation of keratinocytes are key steps of epithelialization. To explore the epithelialization of the wound, immunohistochemical staining was performed to detect the expression of K6 and K1, with K6 being an indicator of keratinocyte hyperproliferation. As Fig. 8A,B showed, five days after treatment, K6 expression levels were significantly higher in the hydrogel + BMSCs group compared with the control and hydrogel groups (p < 0.01, n = 3), whereas no significant differences were observed between the hydrogel and control groups with respect to K6 expression.

K1 is an indicator of terminally differentiated keratinocytes; thus, we compared K1 expression at the re-epithelialized wound sites of the three examined groups 14 and 21 days after treatment. As Fig. 8C,D showed, the expression of K1 was significantly higher in the hydrogel + BMSCs group compared with the control and hydrogel groups (p < 0.01, n = 3) on day 14. By Day 21, significant differences did not exist among the three groups with respect to K1 expression at the re-epithelialized wound site (data not shown).

### Hydrogel + BMSCs combination therapy improved the quality of wound healing

**Figure 9:**
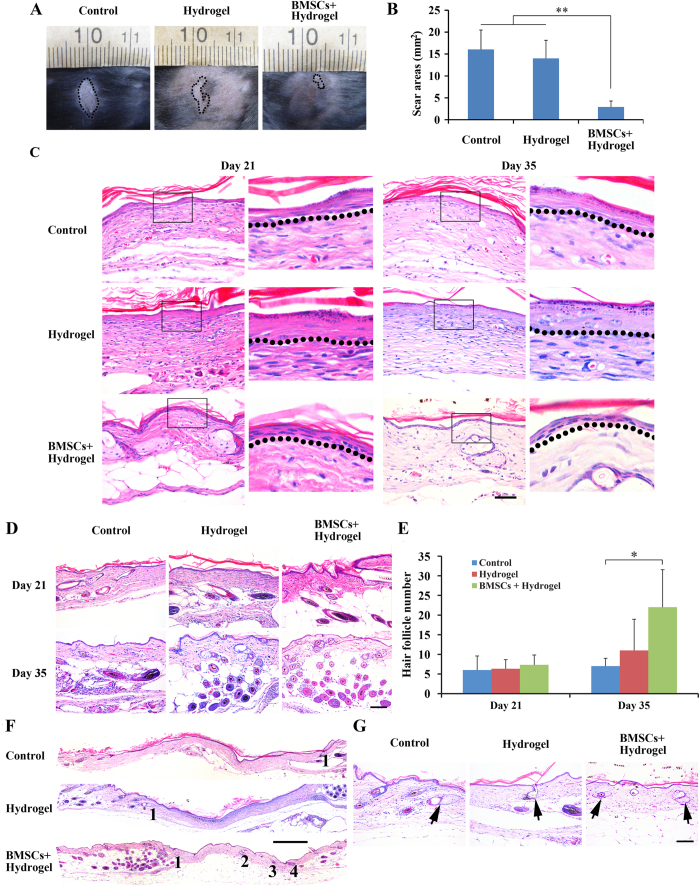
Joint hydrogel + BMSCs treatment enhanced the quality of wound healing. (**A**,**B**) general observations of scar formation in the control, hydrogel, hydrogel + BMSCs group after 35 days treatment (n = 3). (**C**) HE staining showing the structure of the regenerated epidermis in the control, hydrogel, hydrogel + BMSCs group after 21 and 35 days of treatment (n = 3). Scale = 50 μm. (**D**,**E**) HE staining showing the newborn hair follicles at the edge of the wound in the control, hydrogel, hydrogel + BMSCs group after 21 and 35 days of treatment. Scale = 100 μm. (**F**) HE staining showing the distribution of newborn sebaceous glands in the control, hydrogel, hydrogel + BMSCs group after 35 days treatment. Scale = 500 μm. (**G**) HE staining shows the newborn sebaceous glands in the control, hydrogel and hydrogel + BMSCs group after 35 days of treatment. Scale = 100 μm.

Skin wound healing eventually ended with scar formation. As shown in Fig. 9A,B, thirty-five days after treatment, general observations showed that the BMSCs + hydrogel group had a significantly different average scar area compared with the control and hydrogel groups (p < 0.01, n = 3). Additionally, regenerated epidermis was detected. The normal epidermis of type 2 diabetic transgenic mice consists of one or two layers of keratinocytes; here, the arrangement of basal keratinocytes was loose (Figure S1). Figure 9C shows the structure of the epidermis at the midportion of the scar after 21 and 35 days of treatment among the three groups. We found that on both days 21 and 35 after treatment, the structure of the epidermis was similar to that of the epidermis in the BMSCs + hydrogel group. Although there was no easily identifiable basal layer in the control or hydrogel groups, the epidermis was already over-differentiated and without cell nuclei.

We further analysed the structure of the regenerated dermis after 3 and 5 weeks of treatment. As Fig. 9D,E show, we observed an obvious increase in the number of hair follicles at the edge of BMSCs + hydrogel-treated wounds compared with the control and hydrogel groups. Additionally, there were some newborn hair follicles in the center of the wound; however, we could not see newborn hair follicles in the center of the wound in the control or hydrogel groups. Moreover, we observed newborn sebaceous glands at the wound site among the three groups. Figure 9F shows the distribution of the newborn sebaceous glands. In the hydrogel + BMSCs group, the sebaceous glands regenerated not only at the edge of the wound but also in the center, whereas the sebaceous glands regenerated only at the edge of the wound in the control and hydrogel groups. Figure 9G clearly shows the regenerated sebaceous glands of the three groups.

## Discussion

It is generally known that diabetic ulcers do not heal well and that current clinical products cannot achieve desired results. To address this concern, here, we showed that BMSCs could be used to treat these chronic wounds; during the proliferation and remodeling stages of wound healing, we obtained interesting results demonstrating an improved chronic inflammatory microenvironment, which is the first condition of making full use of BMSCs.

Diabetic ulcers are characterized by a chronic inflammatory state that manifests primarily as imbalances in pro- and anti-inflammatory cytokines, which mainly result from macrophages^20^. Macrophages have functional phenotypes that can roughly be divided into two groups as follows: M1 (pro-inflammation) and M2 (pro-healing) macrophages^21^. In chronic wounds, the continuous chronic inflammation mainly caused by M1 macrophages results in wound tissue containing high concentrations of pro-inflammatory cytokines, leading to increased protease secretion at the wound site. Proteases can degrade or inhibit a variety of growth factors^22^,^23^. The hydrogel that we designed was capable of inhibiting M1 macrophage expression; thus, the chronic inflammatory microenvironment was improved. The hydrogel will be beneficial for BMSC differentiation and secretion during wound healing.

TGF-β1 is the strongest known fibrosis-promoting factor, it plays a significant role throughout the entire wound healing process^24^ and it can strongly affect granulation tissue formation, myofibroblast transformation and re-epithelialization. Here, our *in vitro* test showed that the hydrogel can promote BMSC-secreted TGF-β1, and the *in vivo* test showed rapid granulation tissue formation and collagen fiber synthesis in the hydrogel + BMSCs group. TGF-β1 not only promotes infiltration and rapid proliferation of fibroblasts but also stimulates the secretion of collagen, hyaluronic acid and other extracellular matrix components by a variety of cells^25^,^26^. Given these effects, it is unsurprising that superior granulation tissue formation and extracellular matrix secretion were observed in the hydrogel + BMSCs group. Myofibroblasts are responsible for wound contraction and are characterized by expression of α-smooth muscle actin (α-SMA). Previous studies have demonstrated that the transformation of fibroblast into myofibroblast is mainly regulated by TGF-β1^27^. Vaughan *et al*. found that TGF-β1 promoted myofibroblast transformation and α-SMA expression in a dose-dependent manner. Additionally, TGF-β1 enhanced the formation of structural elements, including fibronectin fibrils, vinculin and stress fibres, which are important in myofibroblast contractile force generation and transmission^28^.

TGF-β1 can also regulate the re-epithelialization process by enhancing keratinocyte migration^29^. Hori *et al*. showed that TGF-β1 knockout transgenic mice have a delay in re-epithelialization due to hindered keratinocyte migration^30^. The aforementioned results also corroborated our finding that the hydrogel promoted BMSCs to secrete TGF-β1, resulting in a significant difference in α-SMA expression and re-epitheliazation in the hydrogel + BMSCs group.

bFGF is another key regulatory growth factor in wound healing that is involved in angiogenesis and anti-scar formation. The previous study reported that bFGF stimulates the migration and proliferation of endothelial cells to assemble into blood vessels, thereby promoting localized neovascularization^31^,^32^. In this study, we demonstrated that the hydrogel promoted the secretion of bFGF by BMSCs *in vitro*; therefore, we speculate that the increased expression of bFGF promoted endothelial cell growth. Additionally, it is well known that bFGF is able to reduce scar formation in wound healing. Xie *et al*. also reported that bFGF could improve the quality of wound healing and remarkably alleviate scarring in a rabbit ear model of wound healing, which suggests that bFGF exerted a negative effect on scar formation in wound healing^33^. These results were consistent with our finding that less scarring was found in the hydrogel + BMSCs group; we speculate that this effect is also attributable to bFGF.

In this study, we further investigated the quality of ulcer healing, and interesting results were shown in the hydrogel + BMSCs group, such as intact epidermal structure and hair follicle and sebaceous gland regeneration. Previous studies have reported that BMSCs have the capacity to differentiate into new keratinocytes, sebaceous gland cells and hair follicle cells *in vitro* and *in vivo* and that locally transplanted BMSCs may contribute to forming epidermis, hair follicles and sebaceous glands, significantly enhancing the quality of wound healing^6^. In this study, we not only found several newborn hair follicles and sebaceous glands around regenerated tissue, but also found that, interestingly, newborn hair follicles and sebaceous glands were present at the central site of the scar tissue; we believe this could be attributed to BMSC effects.

## Conclusion

We designed a biocompatible hydrogel that can inhibit chronic inflammation at wound sites of diabetic ulcers, and we used this hydrogel to package BMSCs for transplantation into these non-healing wounds. This treatment contributed to the rapid healing of these wounds by promoting granulation tissue formation, angiogenesis, extracellular matrix secretion, wound contraction, re-epithelialization, regeneration of hair follicles and sebaceous glands, and reduced scar formation. Furthermore, we demonstrated that these effects occur because the hydrogel enhanced BMSC secretion of TGF-β1 and bFGF, thereby producing a series of beneficial biological events. These results provide reliable guidance to treat diabetic ulcers in the clinical setting.

## Materials and Methods

### Hydrogel Preparation

The biodegradable multifunctional crosslinker PAA was synthesised as per our previous report^17^. For the gel, a total of 44 mg of N-isopropylacrylamide (NIPAM, Sigma, St Louis, MO, USA) and 1.6 mg of the poly (amidoamine) (PAA) crosslinker were added to 900 μL of sterile distilled water, and these agents were ultrasonically dissolved. Next, 9 μL of 100 mg/mL ammonium persulfate (APS, Sigma) was added to the solution and was ultrasonically mixed. Finally, 1 μL of N, N, N’, N’-tetramethylethylenediamine (TEMED, Sigma) was added, and the solution was centrifuged for 1 minute and then left standing for 30 min to allow the reactions to occur.

### Morphology and Internal Structure

The morphologies of the polymer before and after gelatin formation were characterized by a digital camera. Then, the surface of the prepared hydrogel was trimmed away while the hydrogel was frozen. The resulting hydrogel sample was lyophilized in a freeze dryer, and the sample’s internal structure was then observed using an electron microscope (Hitachi Science Systems, Japan).

### *In Vitro* Degradation

The freeze-dried hydrogel was obtained with the method described above. The dry weight, W_d_, of each piece was obtained. The hydrogel was then immersed in PBS (0.01 M, pH 7.4) containing 10 mM dithiothreitol (DTT) (Sigma) at 37 °C. The remaining hydrogel was collected after 7, 14 and 21 days of immersion and washed three times in distilled water at 37 °C. The collected samples were then lyophilized, and the remaining weight, W_r_, of each sample was determined. The degradation rate was calculated using Equation (1)^34^. These tests (n = 6) and measurements were repeated three times.


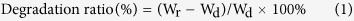


### Isolation and Purification of BMSCs

One healthy 8- to 12-week-old male C57BL/6 mice were purchased from the Laboratory Animal Centre of Southern Medical University. Briefly, bone marrow was obtained as in our previous study and then centrifuged at 1,500 rpm for 5 min. The supernatant was discarded, and the precipitate was re-suspended in complete medium, transferred into a petri dish, and cultured in an incubator at 37 °C and 5% CO_2_^35^. The medium was replaced every 2 days.

### MTT test

The cytotoxicity of PNIPAM-PAA hydrogel was tested using the MTT assay. BMSCs were seeded in PNIPAM-PAA hydrogel-coated 96-well plates at a density of 1 × 10^5^ cells/well and incubated in 5% CO_2_ at 37 °C; a well without PNIPAM-PAA hydrogel coating served as a control. After incubation for 1, 2, and 3 days, the viability of the BMSCs was measured by MTT assay. Before we tested the biocompatibility of this hydrogel, we used dialysis bags (molecular weight cut off, 3000) to dialyse the hydrogel for 3 days to remove the residual NIPAM.

### Immunofluorescence staining

The morphologies of the BMSCs on hydrogels were investigated using immunofluorescence staining. BMSCs were seeded on PNIPAM-PAA hydrogel-coated 96-well plates; at each time point, the cells were washed twice with PBS and fixed with 4% paraformaldehyde solution for 20 min at room temperature. After this, cells were washed twice with PBS and permeabilized using 0.5% Triton X-100 solution for 5 min. Then they were blocked in 5% bovine serum albumin solution for 10 min. Finally, the samples were incubated in phallacidin solution for 20 minutes, washed once and stained with Hoechst for 10 min before inverted fluorescence microscope investigation.

### Cytokine assays

Twenty microliters of sterilized hydrogel was used to coat the bottom of 96-well plates for the hydrogel group; the control group had no hydrogel coating. Fourth-generation BMSCs were seeded at a concentration of 1 × 10^6^ cells/mL in the 96-well plates and cultured for 24 and 72 hours; then, the supernatant was collected. The secretion level of TGF-β1, EGF and bFGF were detected using a commercially available enzyme-linked immunosorbent assay (ELISA) system (Lichen, Shanghai, China) following kit instructions.

### BMSC loading

Fourth-generation BMSCs were digested with 0.25% trypsin, centrifuged, re-suspended in D-Hanks medium, counted, and centrifuged again. A volume of hydrogel was added to reach a cell concentration of 1 × 10^6^ cells/mL, and the resulting solution was mixed gently with a pipette tip^36^. Before using this hydrogel to encapsulate BMSCs, we used dialysis bags (molecular weight cut off, 3000) to dialyse the hydrogel for 3 days to remove the residual NIPAM.

### Wound model

Forty-five 8- to 12-week-old female C57BKS Cg-m+/+ Leprdb type II diabetic mice were purchased from the Model Animal Research Centre of Nanjing University. These mice received an intraperitoneal injection of 0.01 mL 1% pentobarbital sodium (Aladdin, China) per gram of body weight; after the mice were anaesthetised, hair was shaved from both sides of their backs. Next, a skin punch was used to make a circular wound of 8 mm in diameter that excised the full thickness of the skin^37^. After the wound model was established, the mice were randomly divided into three groups, with 15 mice in each group. The wounds of the control mice were left untreated, whereas the wounds of the hydrogel group were filled with 50 μL of hydrogel, and the wounds of the hydrogel + BMSCs group were filled with 50 μL of hydrogel containing 1 × 10^6^ BMSCs/mL. After receiving one treatment, each mouse was individually housed and fed ad libitum. At 0, 5, 7, 14, 21 and 35 days after treatment, a digital camera was used to record the wound of each mouse, and the residual wound area that remained at an early stage of trauma was calculated using Equation (2)^38^. The Bioethics Committee of Southern Medical University approved all animal experiments, which were in accordance with the National Institutes of Health (NIH) Guide for the Care and Use of Laboratory Animals.


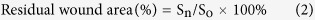


S_0_: initial wound area, S_n_: wound area at different time points, n = 3.

### Histological Observations

On postoperative days 5, 7, 14, 21 and 35, the wound and surrounding tissue (0.5 cm) were carefully excised, rinsed in PBS, and then fixed in 4% Paraformaldehyde (PFA). Samples were dehydrated in a graded ethanol series (70–100%) and embedded in paraffin. Five-micrometer sections were prepared. According to the standard procedures, samples were stained with either Hematoxylin and Eosin (HE) or Masson trichrome, and immunohistochemistry including α-SMA (1:100, Boster), K1 (1:100, Abcam), K6 (1:500, Covance), CD86 and CD163 (1:200, Bioss)^38^.

Image-Pro Plus was used to analyse the average optical density values for K1, K6, α-SMA expression. Five randomly selected fields of view were examined for each group at each time point and used to assess the average optical density value per unit area. Statistics regarding the number of positively stained cells for CD86 and CD163 were obtained using five randomly selected fields of view for each group at each time point.

### Statistical analysis

Statistical analysis was performed using SPSS 13.0 software. Data were expressed as the mean ± S.D. Differences among the groups were assessed using a paired-sample T test or one-way ANOVA. Significance levels were set at *p < 0.05 and **p < 0.01.

## Additional Information

**How to cite this article**: Chen, S. *et al*. Mesenchymal stem cell-laden anti-inflammatory hydrogel enhances diabetic wound healing. *Sci. Rep*. **5**, 18104; doi: 10.1038/srep18104 (2015).

## Supplementary Material

Supplementary Information

## Figures and Tables

**Figure 1:**
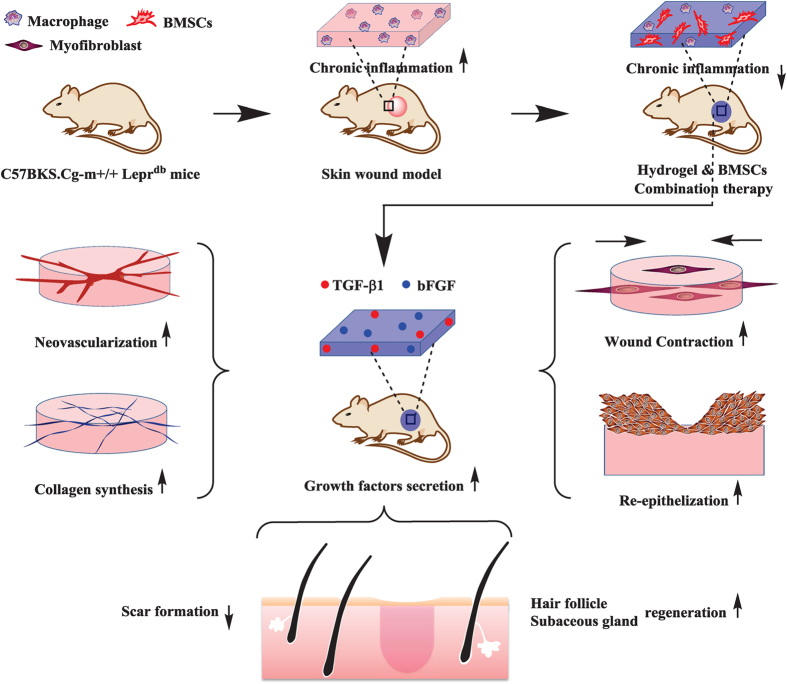
The primary hypothesis of this study. BMSC-laden hydrogels can prohibit chronic inflammation and contribute to growth factor secretion, resulting in accelerated wound contraction, ECM secretion, angiogenesis, re-epithelialization, hair follicle and sebaceous gland regeneration and reduced scar formation.
