# Supplementary material for: Mesenchymal stem cell-laden anti-inflammatory hydrogel enhances diabetic wound healing
Source: Sci Rep. 2015 Dec 8;5:18104. doi: 10.1038/srep18104 (PMC4672289; doi:10.1038/srep18104)
Supplement: Supplementary Information [file srep18104-s1.pdf]

Mesenchymal stem cell-laden anti-inflammatory hydrogel enhances diabetic wound healing

Shixuan Chen [a 1], Junbin Shi [c 1], Min Zhang [a], Yinghua Chen [a], Xueer Wang [a], Lei Zhang [a], Zhihui Tian [a], Yuan Yan [a], Qinglin Li [a], Wen Zhong [d], Malcolm Xing [c]*, Lu Zhang [b]*, Lin Zhang [a]*

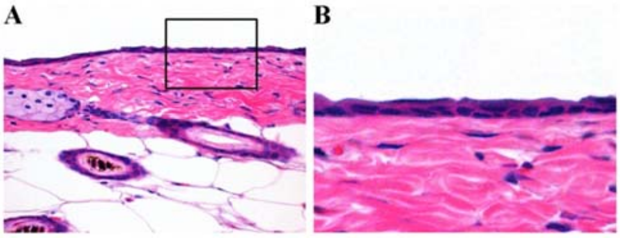

Fig.S1. The normal epidermal structure of type II diabetes mice.
